# Supplementary material for: Identification of poly(ADP-ribose) polymerase 9 (PARP9) as a noncanonical sensor for RNA virus in dendritic cells
Source: Nat Commun. 2021 May 11;12:2681. doi: 10.1038/s41467-021-23003-4 (PMC8113569; doi:10.1038/s41467-021-23003-4)
Supplement: Supplementary file 1 — Supplementary Information [file 41467_2021_23003_MOESM1_ESM.pdf]

Supplementary Information for:

Identification of poly(ADP-ribose) polymerase 9 (PARP9) as a non-canonical sensor for RNA virus in dendritic cells

Junji Xing[1], Ao Zhang[1,2], Yong Du[1], Mingli Fang[1,3], Laurie J. Minze[1], Yong-Jun Liu[4], Xian Chang Li[1,5], Zhiqiang Zhang[1,5,*]

[1]Department of Surgery and Immunobiology and Transplant Science Center, Houston Methodist, Houston, TX 77030, USA
[2]Department of Laboratory Medicine, State Key Laboratory of Oncology in South China, Collaborative Innovation Center for Cancer Medicine, Sun Yat-sen University Cancer Center, Guangzhou 510060, China
[3]Department of Molecular Biology, College of Basic Medical Sciences, Jilin University, Changchun 130021, China
[4]SInnovent Biologics Inc, 168 Dongping St, Suzhou Industry Park, Suzhou 215123, China
[5]Department of Surgery, Weill Cornell Medical College, Cornell University, New York, NY 10065, USA

*Correspondence: zzhang@houstonmethodist.org (Z.Z.)

This document includes Supplementary Figures 1-14, and Supplementary Tables 1-2

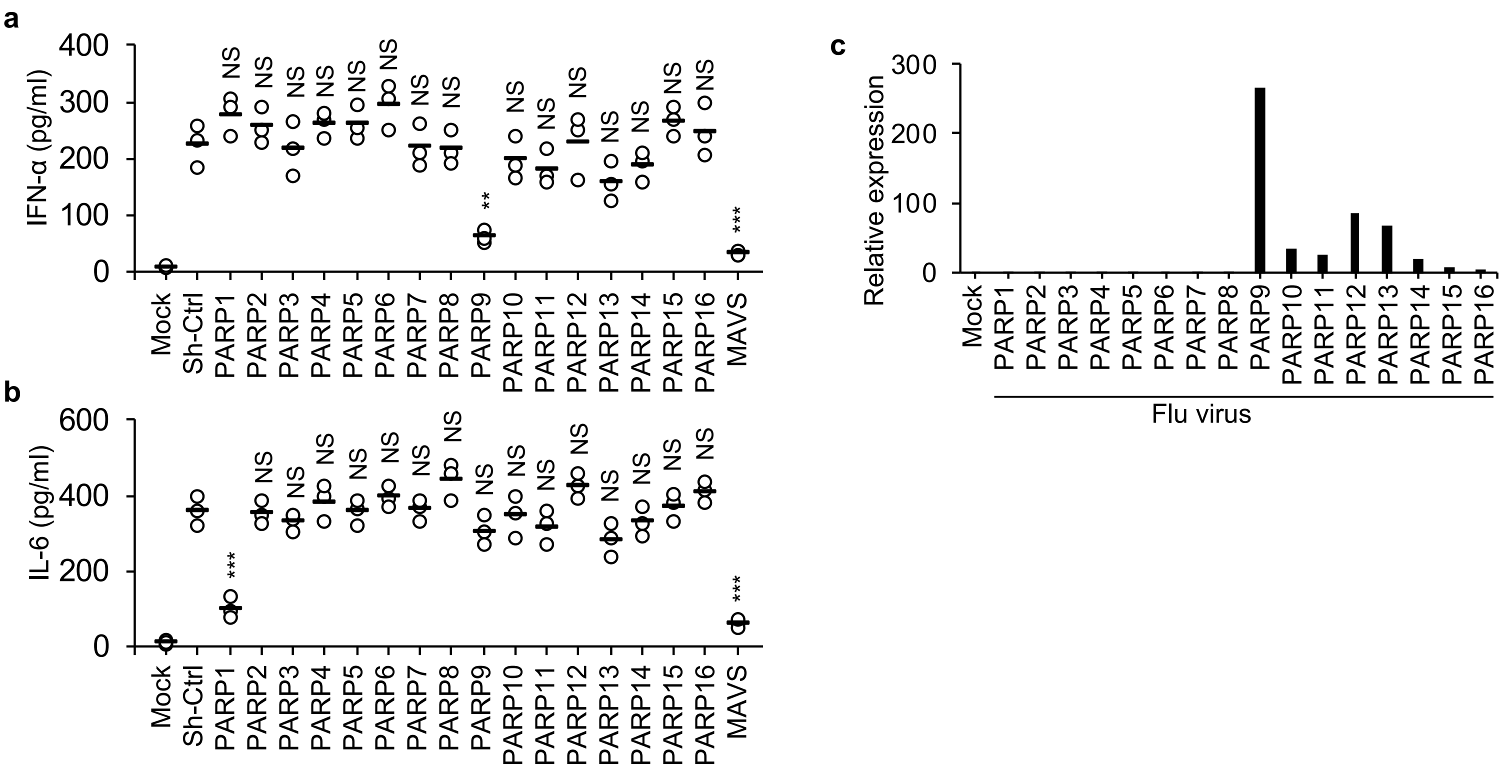

Supplementary Figure 1. Screening of PARPs that modulate the dsRNA polyI:C induced production of type I IFN and proinflammatory cytokine. a,b ELISA of IFN-α (**a**) and IL-6 (**b**) production from THP-1 macrophages treated with the indicated shRNA after a 10 h stimulation with 0.5 μg/ml of long poly I:C delivered by Lipofectamine 3000 (n=3 per group). Each circle represents an individual independent experiment and small solid black lines indicate the average of triplicates. **c**, The profile of PARPs expression in human pDCs purified from PBMCs without and with influenza A virus (influenza A virus PR8 strain, Flu) infection by macroarray. The relative expression of PARPs was compared by plotting the values extracted from the gene expression database. A value < 1 indicated the absence of gene expression. Mock, scrambled shRNA-treated cells without stimulation or pDCs without infection. NS, not significant (p> 0.05), **p< 0.01, ***p< 0.001, p value was calculated by unpaired two-tailed Student's *t* test. Data are from one experiment with duplicate (**c**) or representative of three independent experiments (**a-b**). Exact p values (**a**,p=0.15,p=0.32,p=0.83,p=0.22,p=0.24,p=0.097,p=0.90,p=0.82,p=0.002,p=0.45,p=0.21,p=0.94,p=0.097,p=0.25,p=0.19,p=0.54,p=0.0009; **b**,p=0.0007,p=0.91,p=0.35,p=0.51,p=0.99,p=0.25,p=0.88,p=0.07,p=0.18,p=0.77,p=0.29,p=0.08,p=0.09,p=0.40,p=0.69,p=0.13,p=0.0002).

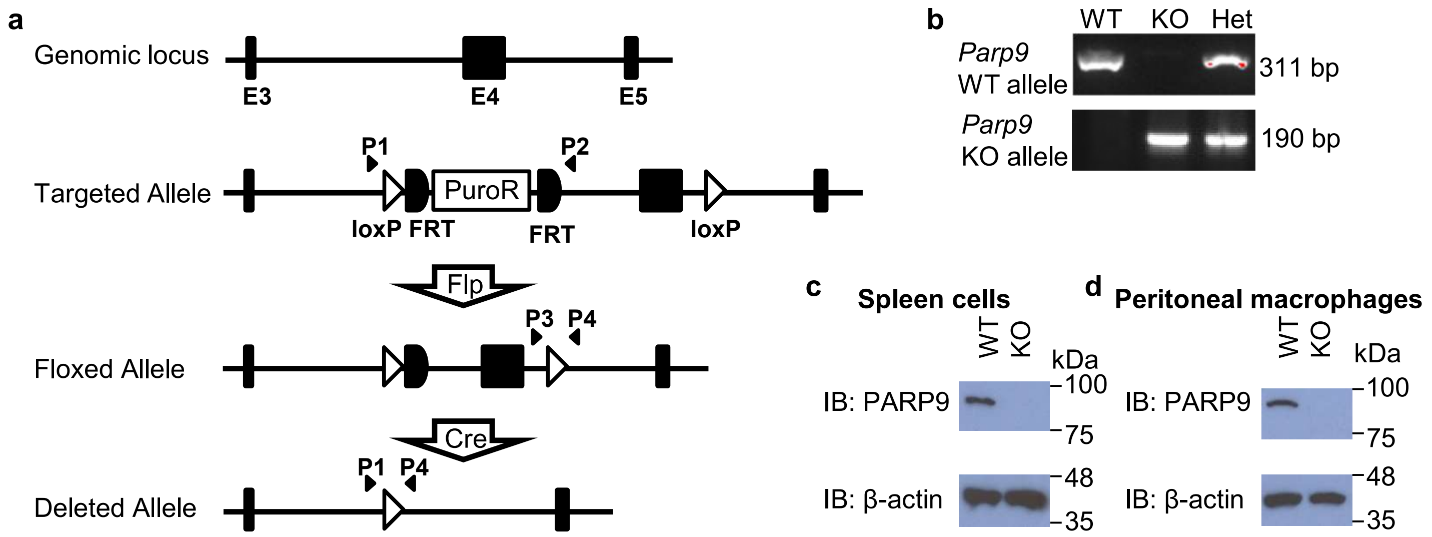

Supplementary Figure 2. *Parp9* gene targeting. a, schematic picture of Parp9 gene targeting using an FRT-LoxP vector, showing the exons 3 to 5 of Parp9 gene. Targeted mice were crossed with FRT deleter (Rosa26-FLPe) mice to generate Parp9-floxed ($Parp9^{fl/fl}$) mice, which were further crossed with EIIa-Cre transgenic mice to generate total knockout (KO) mice. **b**, genotyping PCR analysis of germline Parp9 wild-type (WT), knockout (KO), and heterozygous (Het) mice using P1/P2 primer pair for wild-type allele and P1/P4 primer pair for KO allele. **c,d**, immunoblot (IB) analysis of PARP9 in spleen cells (**c**) and primary peritoneal macrophages (**d**) from wild-type mice (WT) or PARP9 knockout mice (KO). Data are representative of three independent experiments.

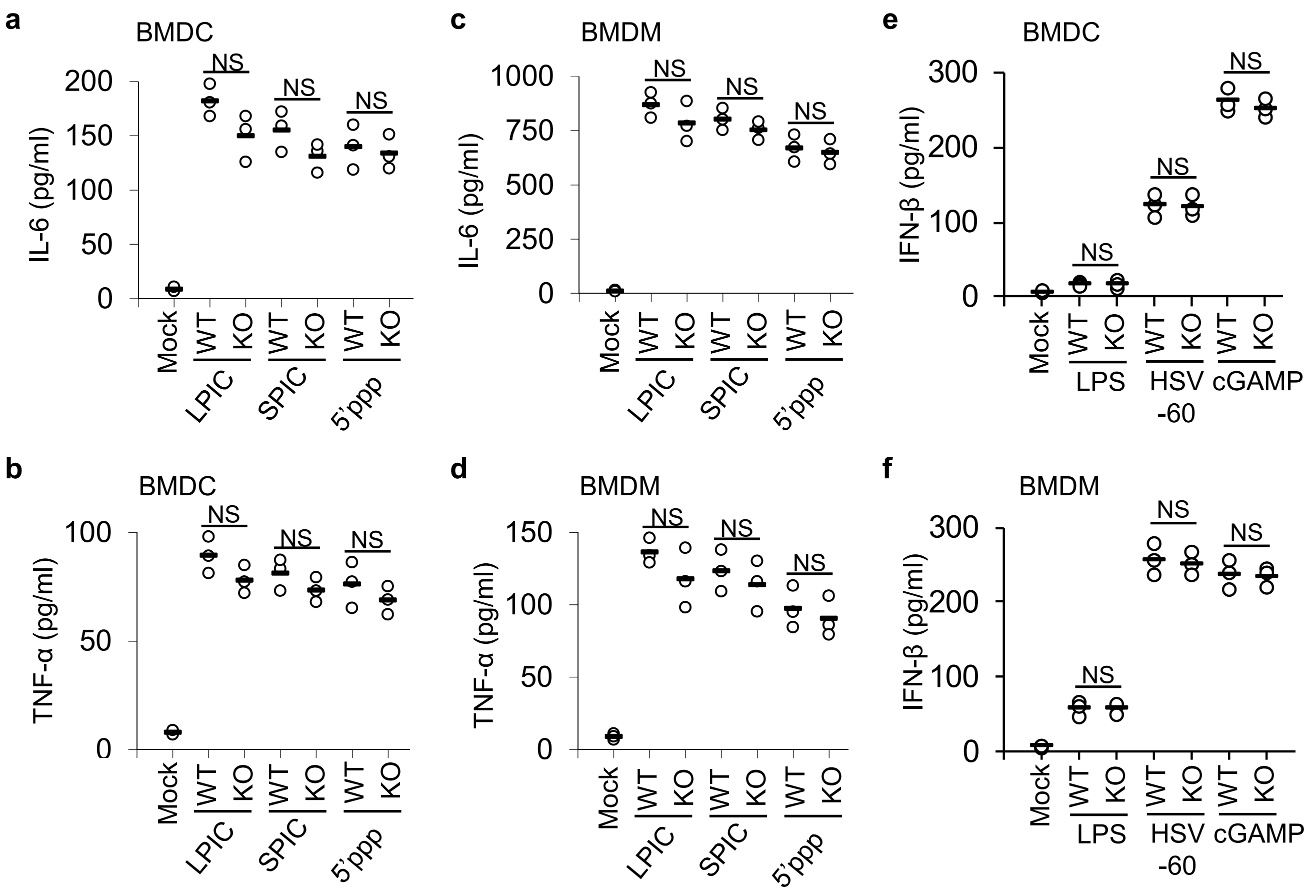

Supplementary Figure 3. PARP9 plays a marginal role to regulate productions of proinflammatory cytokines and IFN-β in response to dsRNA, LPS, dsDNA HSV-60 and cGAMP, respectively. a-f, ELISA of IL-6 (**a,c**) , TNF-α (**b,d**) and IFN-β (**e,f**) production by BMDC (**a,b,e**) or BMDM (**c,d,f**) from wild-type (WT) and *Parp9*$^{-/-}$ (KO) mice after 10 h of stimulation with long poly I:C (LPIC, 0.5 μg/ml), short poly I:C (SPIC, 0.5 μg/ml), 5'pppRNA (5'ppp, 0.5 μg/ml), dsDNA from HSV-1 virus (HSV-60, 2.5 μg/ml) or cGAMP (1.0 μg/ml) delivered by Lipofectamine 3000 or LPS (20 ng/ml) (n=3 per group). Each circle represents an individual independent experiment and small solid black lines indicate the average of triplicates. NS, not significant, and p value was calculated by unpaired two-tailed Student's *t* test. Mock, wild-type BMDC or BMDM without stimulation. Data are representative of three independent experiments. Exact p values (**a**,p=0.102,p=0.146,p=0.71; **b**,p=0.136,p=0.218,p=0.357; **c**,p=0.254,p=0.27,p=0.687; **d**,p=0.224,p=0.503,p=0.578; **e**,p=0.96,p=0.98,p=0.47; **f**,p=0.98,p=0.73,p=0.83).

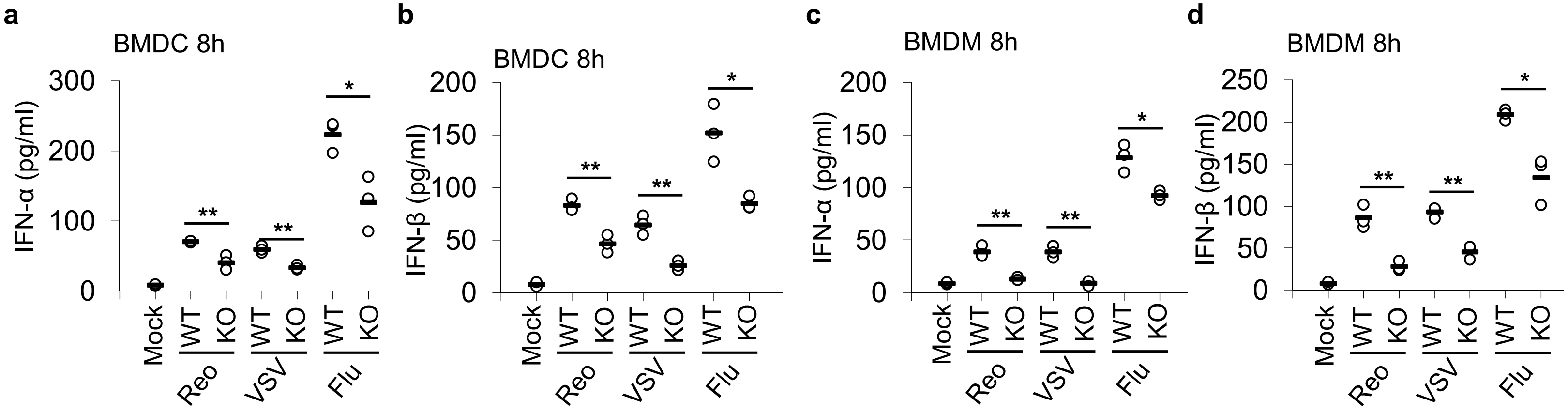

Supplementary Figure 4. PARP9 positively regulates the type I IFN production in response to RNA viruses. a-d, ELISA of IFN-α (**a,c**) and IFN-β (**b,d**) production by BMDC (**a,b**) or BMDM (**c,d**) from wild-type (WT) and *Parp9*$^{-/-}$ (KO) mice after 8 h of infection with Reovirus (Reovirus type 3 strain dearing T3D, Reo), VSV (Vesicular stomatitis virus Indiana strain, VSV) or influenza A virus (influenza A virus PR8 strain, Flu) (n=3 per group). The viruses were used at an MOI of 5. Each circle represents an individual independent experiment and small solid black lines indicate the average of triplicates. *p< 0.05, **p< 0.01, p value was calculated by unpaired two-tailed Student's *t* test. Mock, wild-type BMDC or BMDM without infection. Data are representative of three independent experiments. Exact p values (**a**,p=0.0078,p=0.0018,p=0.02; **b**,p=0.0034,p=0.0028,p=0.014; **c**,p=0.0014,p=0.0011,p=0.011; **d**,p=0.0026,p=0.0015,p=0.012).

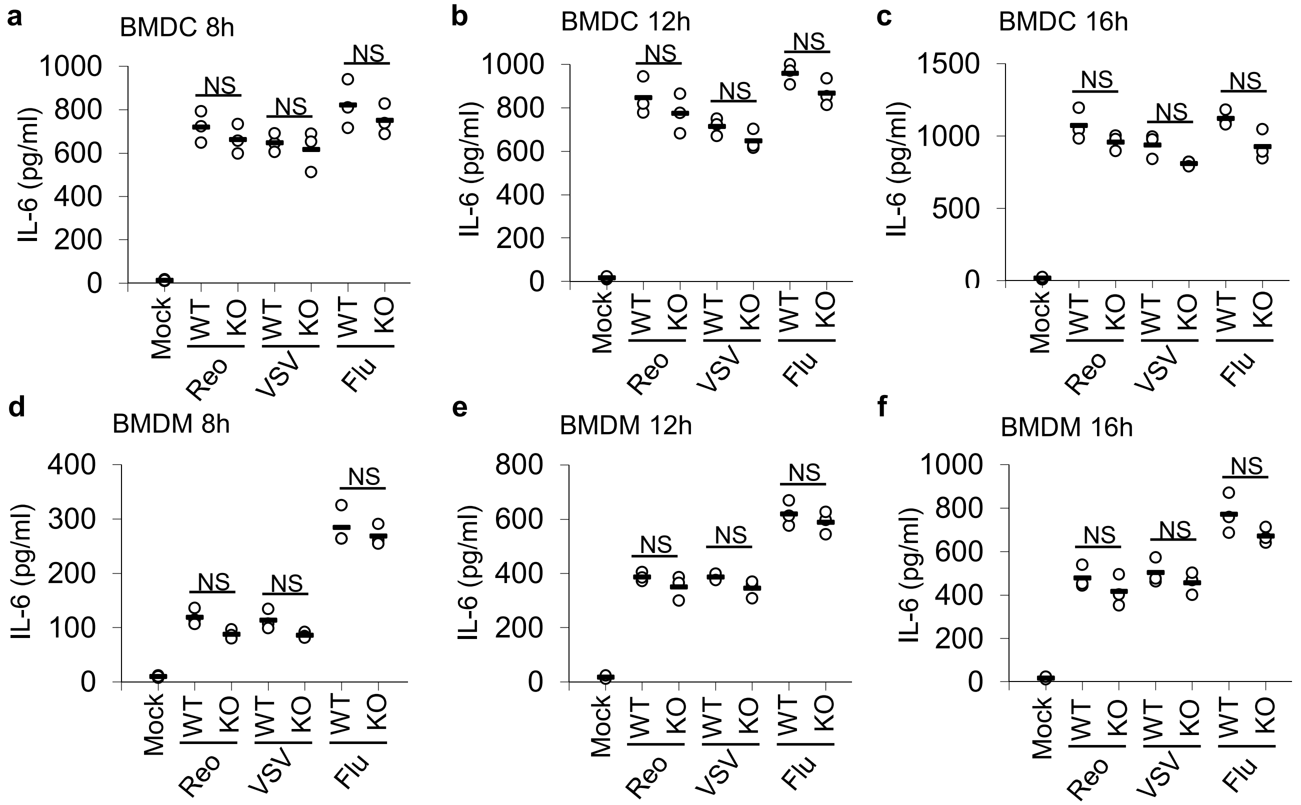

Supplementary Figure 5. PARP9 plays a marginal role to regulate IL-6 production in response to RNA viruses. a-c, ELISA of IL-6 production by BMDC from wild-type (WT) and *Parp9*$^{-/-}$ (KO) mice after 8h (**a**) , 12h (**b**) or 16h (**c**) of infection with Reovirus (Reovirus type 3 strain dearing T3D, Reo), VSV (Vesicular stomatitis virus Indiana strain, VSV) or influenza A virus (influenza A virus PR8 strain, Flu) (n=3 per group). **d-f**, ELISA of IL-6 production by BMDM from wild-type (WT) and *Parp9*$^{-/-}$ (KO) mice after 8h (**d**) , 12h (**e**) or 16h (**f**) of infection with Reovirus, VSV or Flu virus (n=3 per group). The viruses were used at an MOI of 5. Each circle represents an individual independent experiment and small solid black lines indicate the average of triplicates. NS, not significant, and p value was calculated by unpaired two-tailed Student's *t* test. Mock, wild-type BMDCs or BMDMs without infection. Data are representative of three independent experiments. Exact p values (**a**,p=0.37,p=0.64,p=0.41; **b**,p=0.37,p=0.14,p=0.11; **c**,p=0.19,p=0.06,p=0.055; **d**,p=0.057,p=0.07,p=0.54; **e**,p=0.25,p=0.103,p=0.46; **f**,p=0.302,p=0.37,p=0.16).

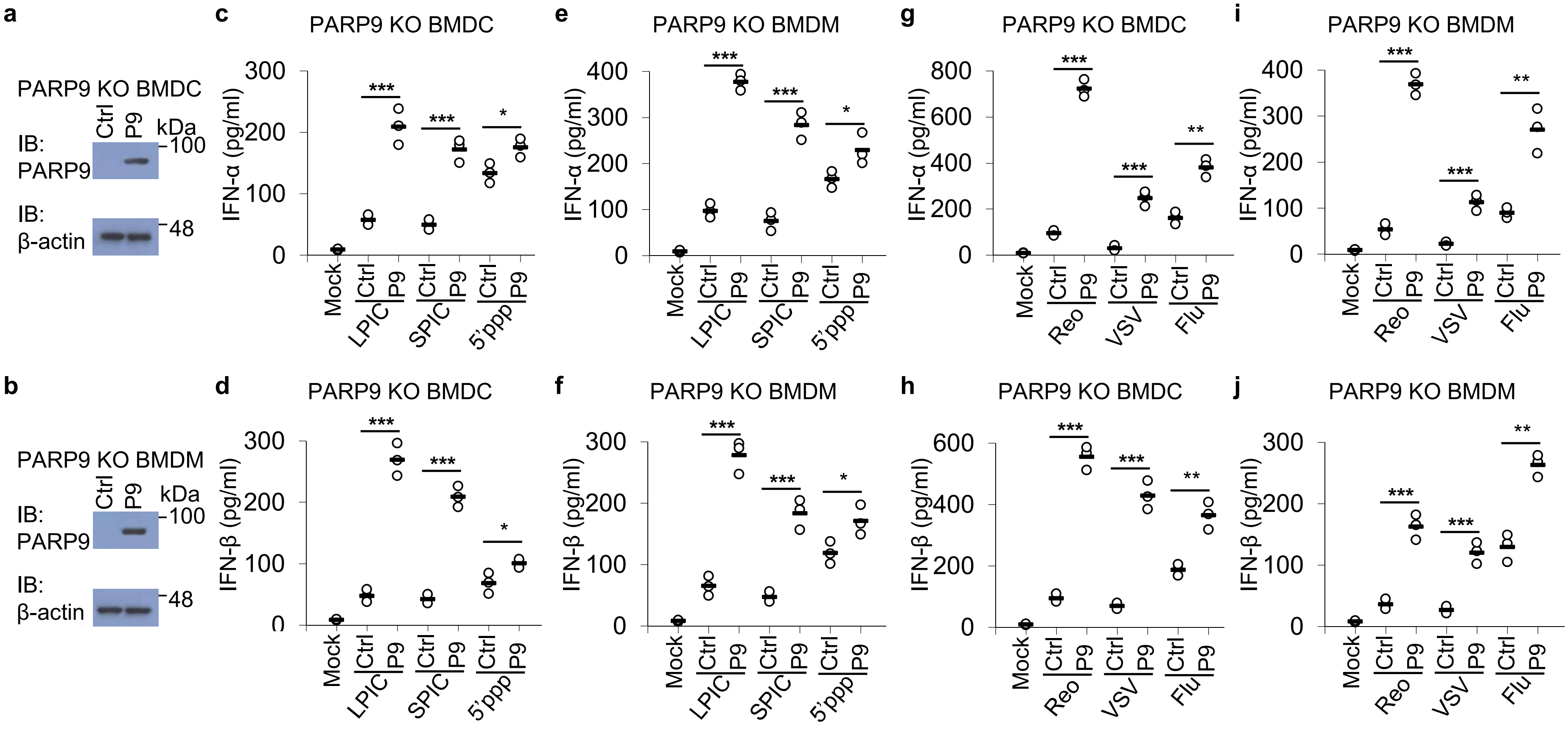

Supplementary Figure 6. Overexpression of PARP9 rescues the type I IFN production in response to dsRNA and RNA viruses in PARP9 KO BMDC and BMDM. a,b, immunoblot (IB) analysis of PARP9 expression in PARP9 KO BMDC (**a**) or BMDM (**b**) reconstituted with control vector (Ctrl) or wild-type PARP9 (P9). **c-f**, ELISA of IFN-α (**c,e**) and IFN-β (**d,f**) production by PARP9 KO BMDC (**c,d**) or BMDM (**e,f**) reconstituted with control vector (Ctrl) or wild-type PARP9 (P9) after 10 h of stimulation with long poly I:C (LPIC, 0.5 μg/ml), short poly I:C (SPIC, 0.5 μg/ml) and 5'pppRNA (5'ppp, 0.5 μg/ml) delivered by Lipofectamine 3000 (n=3 per group). **g-j**, ELISA of IFN-α (**g,i**) and IFN-β (**h,j**) production by PARP9 KO BMDC (**g,h**) or BMDM (**i,j**) reconstituted with control vector (Ctrl) or wild-type PARP9 (P9) after 12 h of infection with Reovirus (Reo), VSV or influenza A virus (Flu) (n=3 per group). The viruses were used at an MOI of 5. Each circle represents an individual independent experiment and small solid black lines indicate the average of triplicates for results in (**c-j**). $^*p < 0.05$, $^{**}p < 0.01$, and $^{***}p < 0.001$, p value was calculated by unpaired two-tailed Student's *t* test. Mock, PARP9 KO BMDC or BMDM without stimulation or infection. Data are representative of three independent experiments. Exact p values (**c**,p=0.00099,p=0.0005,p=0.049; **d**,p=0.00017,p=0.0001,p=0.048; **e**,p=0.00003,p=0.00058,p=0.046; **f**,p=0.0003,p=0.0008,p=0.042; **g**,p=0.00001,p=0.0004,p=0.0014; **h**,p=0.00004,p=0.0002,p=0.003; **i**,p=0.00003,p=0.00094,p=0.0034; **j**,p=0.0006,p=0.0009,p=0.0013).

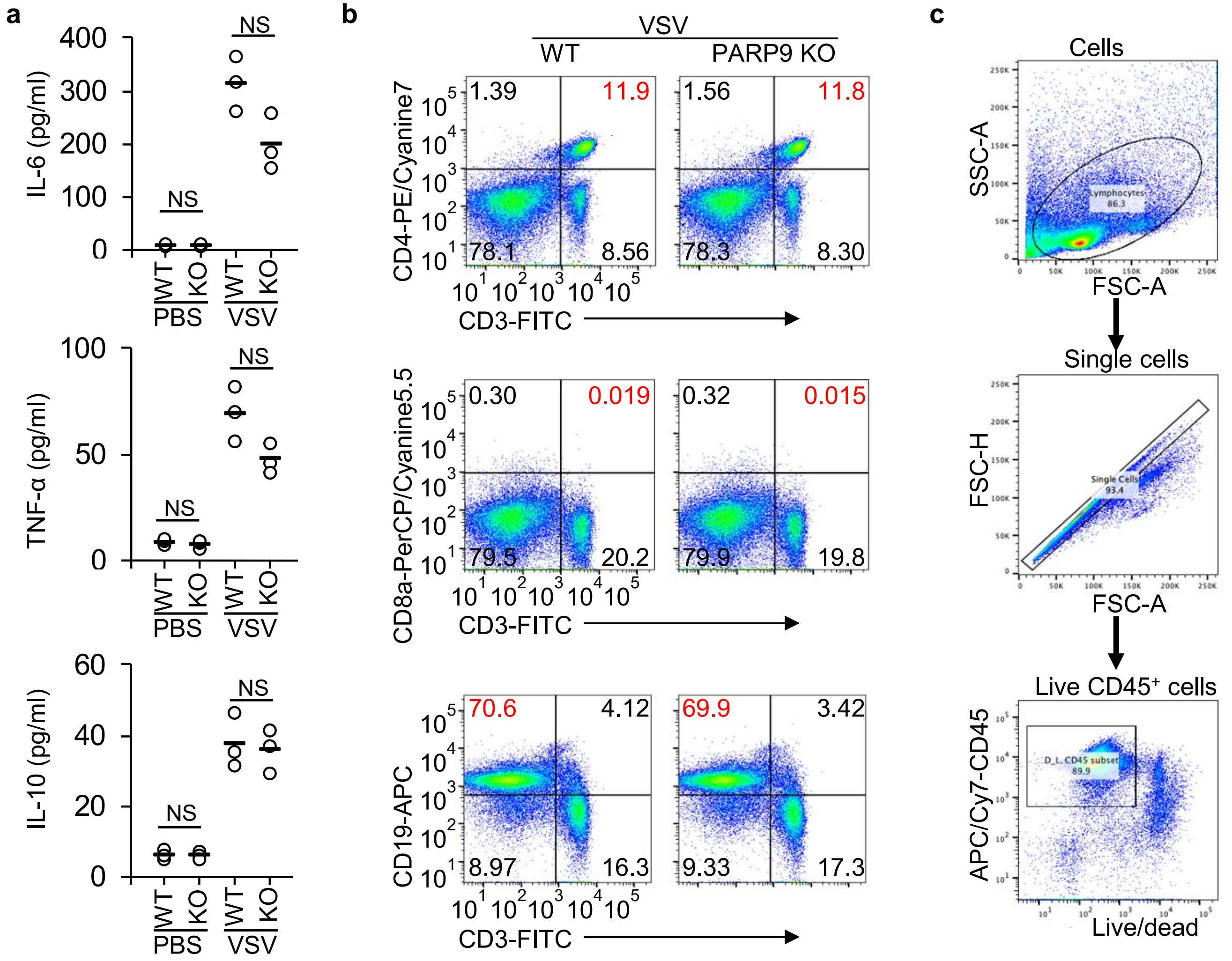

Supplementary Figure 7. PARP9 does not affect the adaptive immunity and production of inflammatory cytokines IL-6, TNF-α and IL-10 after VSV infection *in vivo*. **a**, ELISA of IL-6, TNF-α and IL-10 in sera from *PARP9*$^{+/+}$ (WT) and *PARP9*$^{-/-}$ (KO) mice (three per group) infected for 24 h by intraperitoneal injection of phosphate-buffered saline (PBS) or VSV (5×10^7 PFU per mouse) (n=3 per group). Each circle represents an individual independent experiment and small solid black lines indicate the average of triplicates. **b**, Flow cytometry analysis of $CD4^+$, $CD8^+$ and B cells from *PARP9*$^{+/+}$ (WT) and *PARP9*$^{-/-}$ (KO) mice infected for 24 h by intraperitoneal injection of VSV (5×10^7 PFU per mouse) using CD3-FITC, CD4-PE/Cyanine7, CD8a-PerCP/Cyanine5.5, and CD19-APC antibodies. **c**, Representative FACS plots showing the gating strategy for analyzing T and B cell population in spleen of the mice. Flow cytometry data were acquired on a LSR-II flow cytometer (Beckton Dickinson) and analyzed using FlowJo v10 software (Tree Star). NS, not significant, and p value was calculated by unpaired two-tailed Student's *t* test. Data are representative of three independent experiments. Exact p values (**a**,upper, p=0.84,p=0.051; middle, p=0.47,p=0.06; lower, p=0.77,p=0.76).

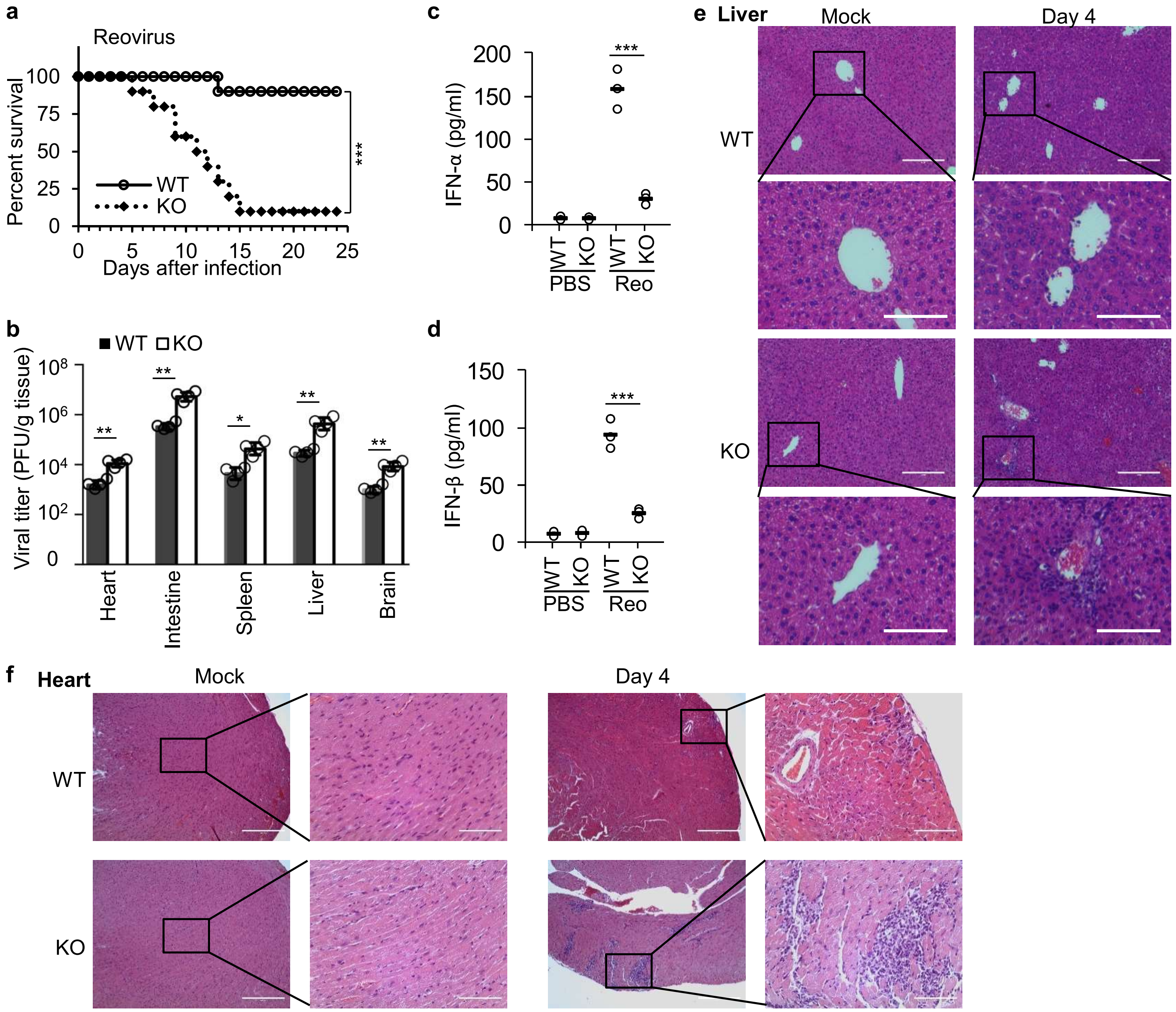

a
Reovirus
Percent survival
100
75
50
25
0
WT
KO
***
0
5
10
15
20
25
Days after infection
b
WT
KO
Viral titer (PFU/g tissue)
10^8
10^6
10^4
10^2
0
**
**
*
**
**
Heart
Intestine
Spleen
Liver
Brain
c
IFN-α (pg/ml)
200
150
100
50
0
***
WT
KO
WT
KO
PBS
Reo
d
IFN-β (pg/ml)
150
100
50
0
***
WT
KO
WT
KO
PBS
Reo
e Liver
Mock
Day 4
WT
KO
f Heart
Mock
Day 4
WT
KO

Supplementary Figure 8. PARP9 plays an important role in host defense against reovirus infection in vivo. a, survival of age- and sex-matched *PARP9*$^{+/+}$ mice (WT) and *PARP9*$^{-/-}$ mice (KO) after intraperitoneal infection with reovirus (5×10^7 PFU per mouse) (n=10 per group). **b**, plaque assay of reovirus titers in the heart, intestine, spleen, liver, and brain of *PARP9*$^{+/+}$ mice (WT) and *PARP9*$^{-/-}$ mice (KO) infected for 2 days by intraperitoneal infection of reovirus (n=4 per group). Error bars indicate standard error of the mean. **c,d**, ELISA of and IFN-α (**c**) and IFN-β (**d**) in heart homologates from mice (n=3 per group) as in **b**. Each circle represents an individual independent experiment and small solid black lines indicate the average of triplicates. **e, f**, hematoxylin and eosin (H&E)-staining of liver (**e**) and heart (**f**) sections from WT and PARP KO mice left infected (Mock) or infected for 4 days by intraperitoneal infection of reovirus. Scale bars represent 200 μm (**e**) or 400 μm (**f**) for original images and 50μm (**e**) or 100 μm (**f**) for enlarged images. *p< 0.05, **p< 0.01, and ***p< 0.001, p value was calculated by unpaired two-tailed Student's *t* test and Gehan-Breslow-Wilcoxon test for survival analysis. Data are representative of three independent experiments. Exact p values (**a**,p=0.0006; **b**,p=0.0051,p=0.0015,p=0.016,p=0.004,p=0.004; **c**,p=0.0008; **d**,p=0.00096).

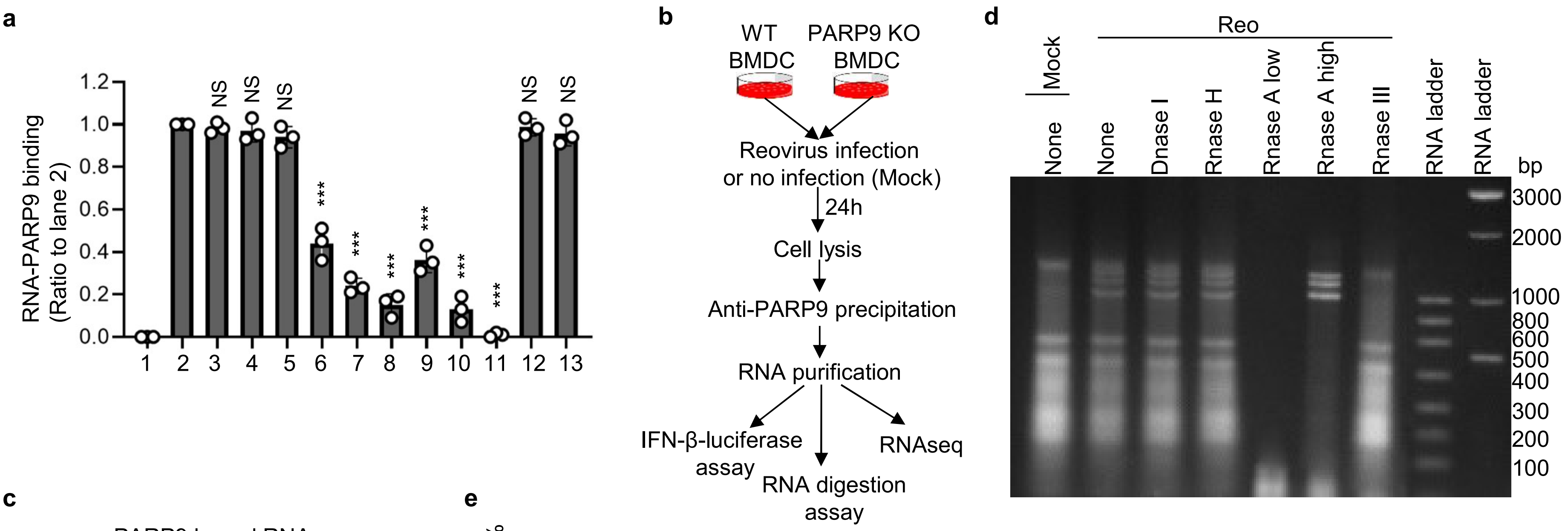

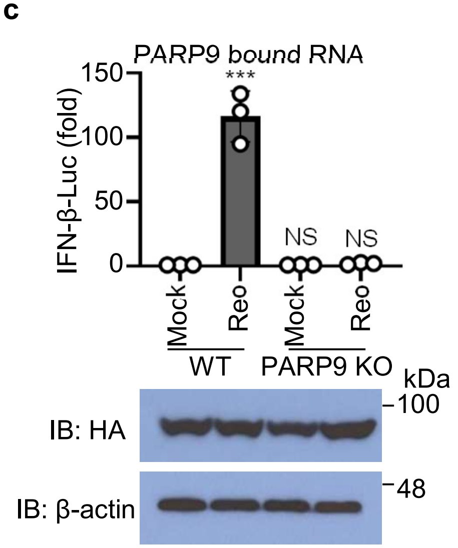

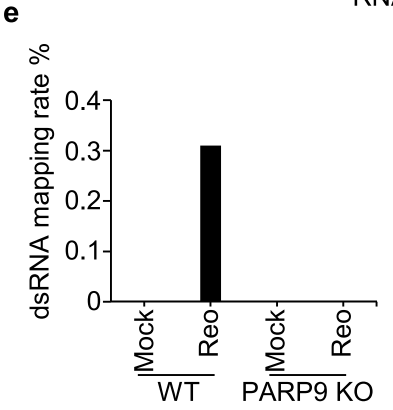

f

Name	Reovirus gene	Start	End
**Reo1187**	S4	8	1187
**Reo1198**	S3	11	1198
**Reo1320**	S2	16	1320
**Reo1410**	S1	9	1410

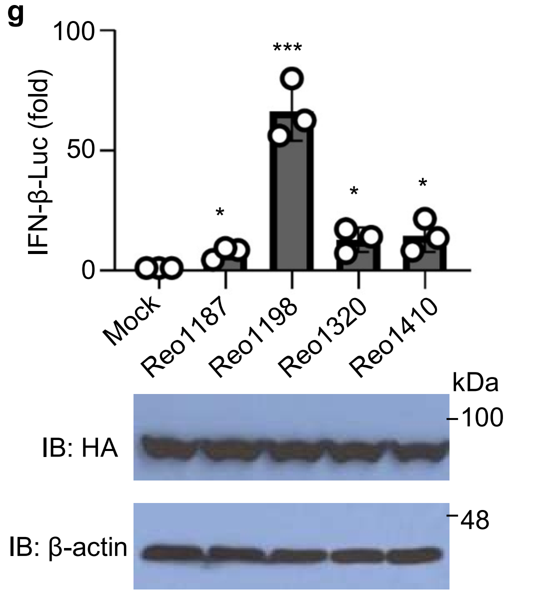

Supplementary Figure 9. The natural ligand of PARP9 is viral dsRNA. a, Quantification of RNA and PARP9 binding activity in Fig. 4c expressed as the ratio of RNA-PARP9 binding relative to lane 2 using the densitometric analysis by ImageJ software (n=3 per group). **b**, experimental setup for the isolation and identification of RNAs from PARP9 precipitates of WT and PARP9 KO BMDC; this figure is created by author Junji Xing. **c**, IFN-β-luciferase reporter activity and immunoblot (IB) analysis of HA-tagged PARP9 expression in HEK 293T cells overexpressed with HA-PARP9, and then transfected for 16h with 10 μl RNA from PARP9 precipitates of WT and PARP9 KO BMDC left uninfected (Mock) or infected with Reovirus at MOI of 20, isolated as in (**b**); results are presented relative to those of PARP9 precipitates from uninfected BMDC, set as 1 (n=3 per group). **d**, enzymatic digestion of RNA from PARP9 precipitates of WT BMDC left uninfected (Mock) or infected with Reovirus (Reo) by no treatment (None) or treatment with Dnase I, Rnase H, Rnase A with low (10mM NaCl) or high (1M NaCl) salt concentrations and Rnase III. **e**, percent of dsRNA mapping rate bound by PARP9 in WT and PARP9 KO BMDC left uninfected (Mock) or infected with Reovirus. **f**, the viral dsRNA sequence bound by PARP9 in WT BMDC infected with Reovirus by RNAseq analysis. **g**, IFN-β-luciferase reporter activity and immunoblot (IB) analysis of HA-tagged PARP9 expression in HEK 293T cells overexpressed with HA-PARP9, and then left transfected (Mock) or transfected for 10h with dsRNA Reo1187, Reo1198, Reo1320 and Reo1410 (1 μg/ml) delivered by Lipofectamine 3000 (n=3 per group). Error bars indicate standard error of the mean for results in (**a,c,g**). NS, not significant ($p > 0.05$), *$p < 0.05$, ***$p < 0.001$, p value was calculated by unpaired two-tailed Student's *t* test. Data are from one experiment with duplicate (**e,f**) or representative of three independent experiments (**a,c-d,g**). Exact p values (**a**,p=0.31,p=0.38,p=0.11,p=0.0002,p<0.00001,p<0.00001,p=0.00006,p=0.00001,p<0.00001,p=0.598,p=0.26; **c**,p=0.0005,p=0.51,p=0.098; **g**,p=0.014,p=0.0008,p=0.016,p=0.025).

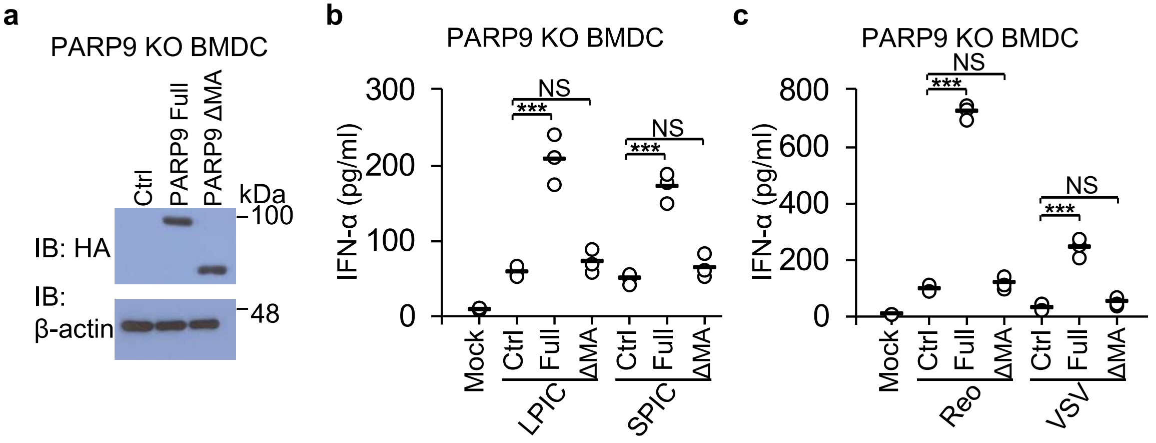

Supplementary Figure 10. Overexpression of full PARP9, but not mutant PARP9 ΔMA, rescues IFN-α production in response to dsRNA and RNA viruses in PARP9 KO BMDC. a, immunoblot (IB) analysis of HA-tagged PARP9 expression in PARP9 KO BMDC reconstituted with control vector (Ctrl), wild-type PARP9 (PARP9 Full) or its mutant PARP9 ΔMA. **b,c**, ELISA of IFN-α production by PARP9 KO BMDC reconstituted with control vector (Ctrl), wild-type PARP9 (Full) or its mutant PARP9 (ΔMA) after 10 h of stimulation with long poly I:C (LPIC, 0.5 μg/ml) and short poly I:C (SPIC, 0.5 μg/ml) delivered by Lipofectamine 3000 (**b**), or after 12 h of infection with Reovirus (Reo) and VSV (**c**) (n=3 per group). The viruses were used at an MOI of 5. Each circle represents an individual independent experiment and small solid black lines indicate the average of triplicates. NS, not significant (p> 0.05) and **p< 0.01, p value was calculated by unpaired two-tailed Student's *t* test. Mock, PARP9 KO BMDC without stimulation or infection. Data are representative of independent experiments. Exact p values (**b**,p=0.00095,p=0.247,p=0.0005,p=0.197; **c**,p<0.00001,p=0.242,p=0.0004,p=0.176).

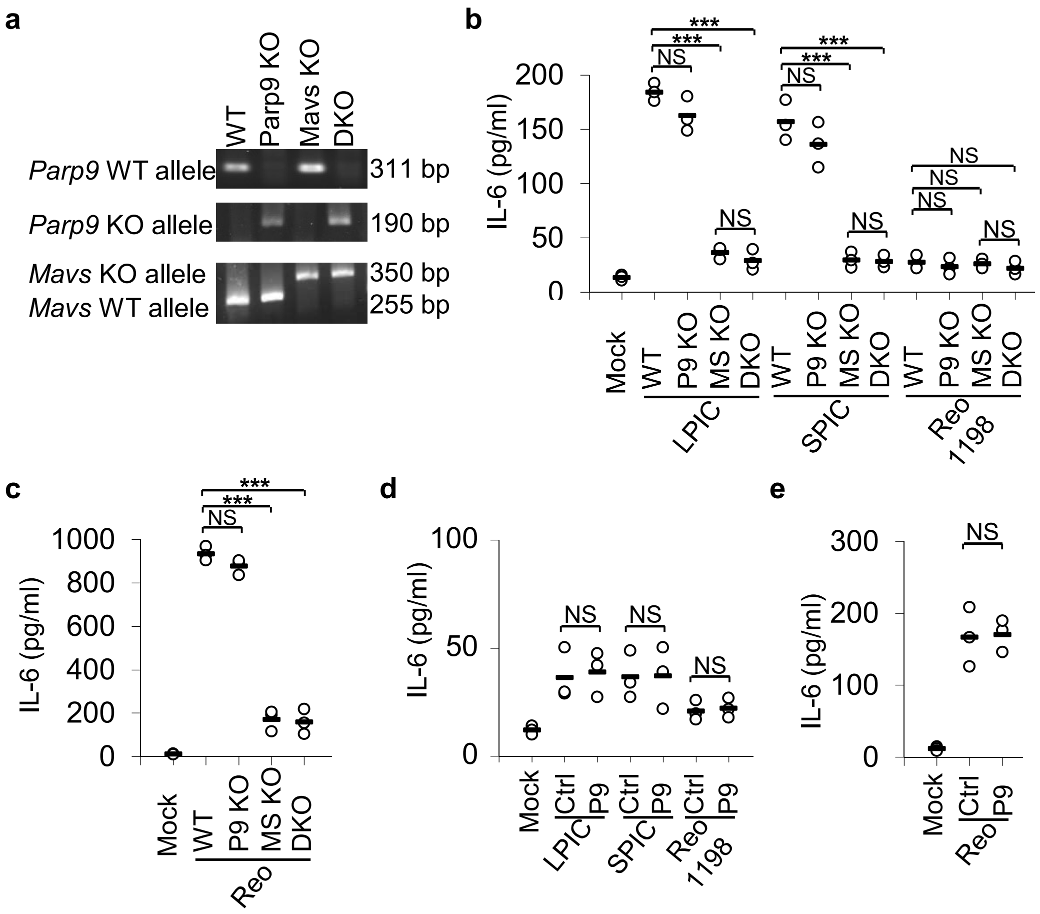

Supplementary Figure 11. Knockout or overexpression of PARP9 does not affect the IL-6 production in BMDC in response to dsRNA and RNA virus. a, genotyping PCR analysis of wild-type (WT), Parp9 knockout (KO), Mavs KO and Parp9/Mavs double KO (DKO) mice using primer pairs for wild-type allele and KO allele of Parp9 gene and Mavs gene, respectively. **b,c**, ELISA of IL-6 production in BMDC from wild-type (WT), PARP9 knockout (P9 KO), MAVS knockout (MS KO) and PARP9/MAVS double knockout (DKO) mice after after 10 h of stimulation with long poly I:C (LPIC, 0.5 μg/ml), short poly I:C (SPIC, 0.5 μg/ml) and Reo1198 dsRNA (Reo1198, 1 μg/ml) delivered by Lipofectamine 3000 (**b**), or after 12h of infection with Reovirus (Reo) at MOI of 5 (**c**) (n=3 per group). **d,e**, ELISA of IL-6 production in PARP9/MAVS double knockout (DKO) BMDC reconstituted with control vector (Ctrl) or wild-type PARP9 (P9) after 10 h of stimulation with long poly I:C (LPIC, 0.5 μg/ml), short poly I:C (SPIC, 0.5 μg/ml) and Reo1198 dsRNA (Reo1198, 1 μg/ml) delivered by Lipofectamine 3000 (**d**), or after 12h of infection with Reovirus (Reo) at MOI of 5 (**e**) (n=3 per group). Each circle represents an individual independent experiment and small solid black lines indicate the average of triplicates. NS, not significant (p> 0.05), ***p< 0.001, p value was calculated by unpaired two-tailed Student's *t* test. Mock, BMDC without stimulation or infection. Data are representative of three independent experiments. Exact p values (**b**,p=0.107,p=0.00002,p=0.00005,p=0.31,p=0.26,p=0.0008,p=0.0009,p=0.57,p=0.52,p=0.77,p=0.35,p=0.41; **c**,p=0.12,p=0.00002,p=0.00003; **d**,p=0.72,p=0.68,p=0.29; **e**,p=0.90).

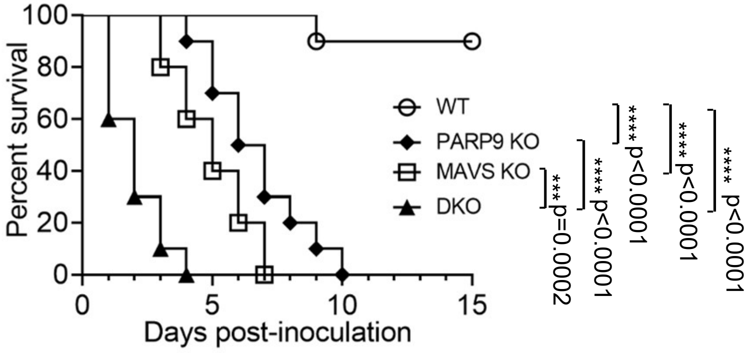

Supplementary Figure 12. Susceptibility comparison of wild-type, PARP9 KO, MAVS KO and PARP9/MAVS double knockout (DKO) mice to VSV infection *in vivo*. Survival of age- and sex-matched *PARP9*$^{+/+}$ (WT), *PARP9*$^{-/-}$ (PARP9 KO), *MAVS* knockout (MAVS KO) and *PARP9/MAVS* double knockout (DKO) mice after intraperitoneal infection with VSV (1×10^6 PFU per mouse) (n=10 per group). ***p< 0.001, ***p< 0.0001, p value was calculated by a two sides Gehan-Breslow-Wilcoxon test and provided on the graph. Data are representative of three independent results.

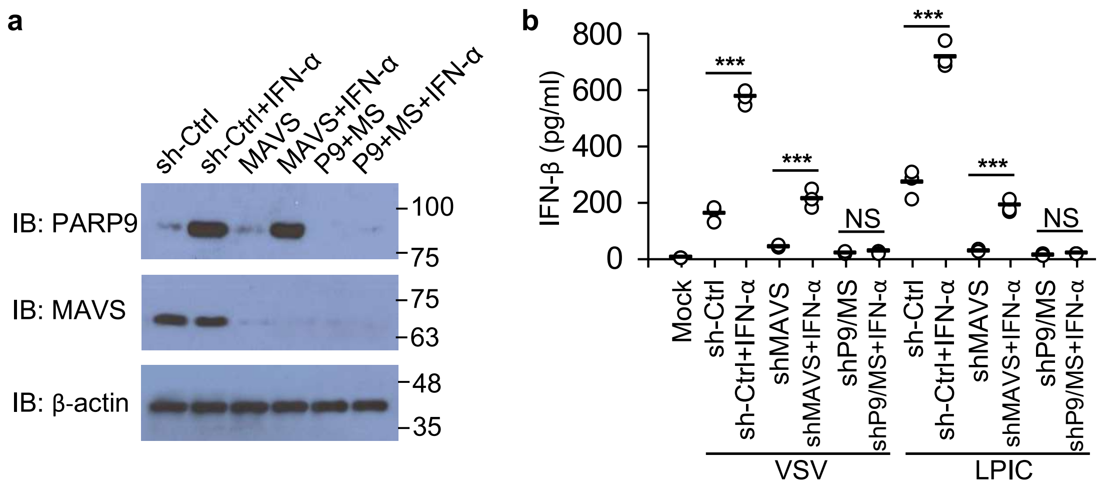

Supplementary Figure 13. PARP9 initiates and amplifies MAVS-independent production of type I IFN in human MDDC. a, Immunoblot analysis of PARP9 or MAVS in human monocyte-derived dendritic cells (MDDC) treated with shRNA to knockdown expression of MAVS or both PARP9 and MAVS (P9/MS), and treated with IFN-α (20 pg/ml) for 2h. A scrambled shRNA served as a control (sh-Ctrl). The β-Actin served as the loading control. **b**, ELISA of IFN-β production in human MDDC treated with the indicated shRNA and IFN-α (20 pg/ml) for 2h after 10 h of stimulation with long poly I:C (LPIC, 0.5 μg/ml) delivered by Lipofectamine 3000 or VSV infection (n=3 per group). The VSV was used at an MOI of 5. Individual circles represent the value from each independent experiment and small solid black lines indicate the average of triplicates. NS, not significant, ***$P<0.001$, p value was calculated by unpaired two-tailed Student's *t* test. Mock, human MDDC without stimulation or infection. Data are representative of three independent experiments. Exact p values (**b**,p=0.00007,p=0.00065,p=0.42,p=0.00035,p=0.00033,p=0.098).

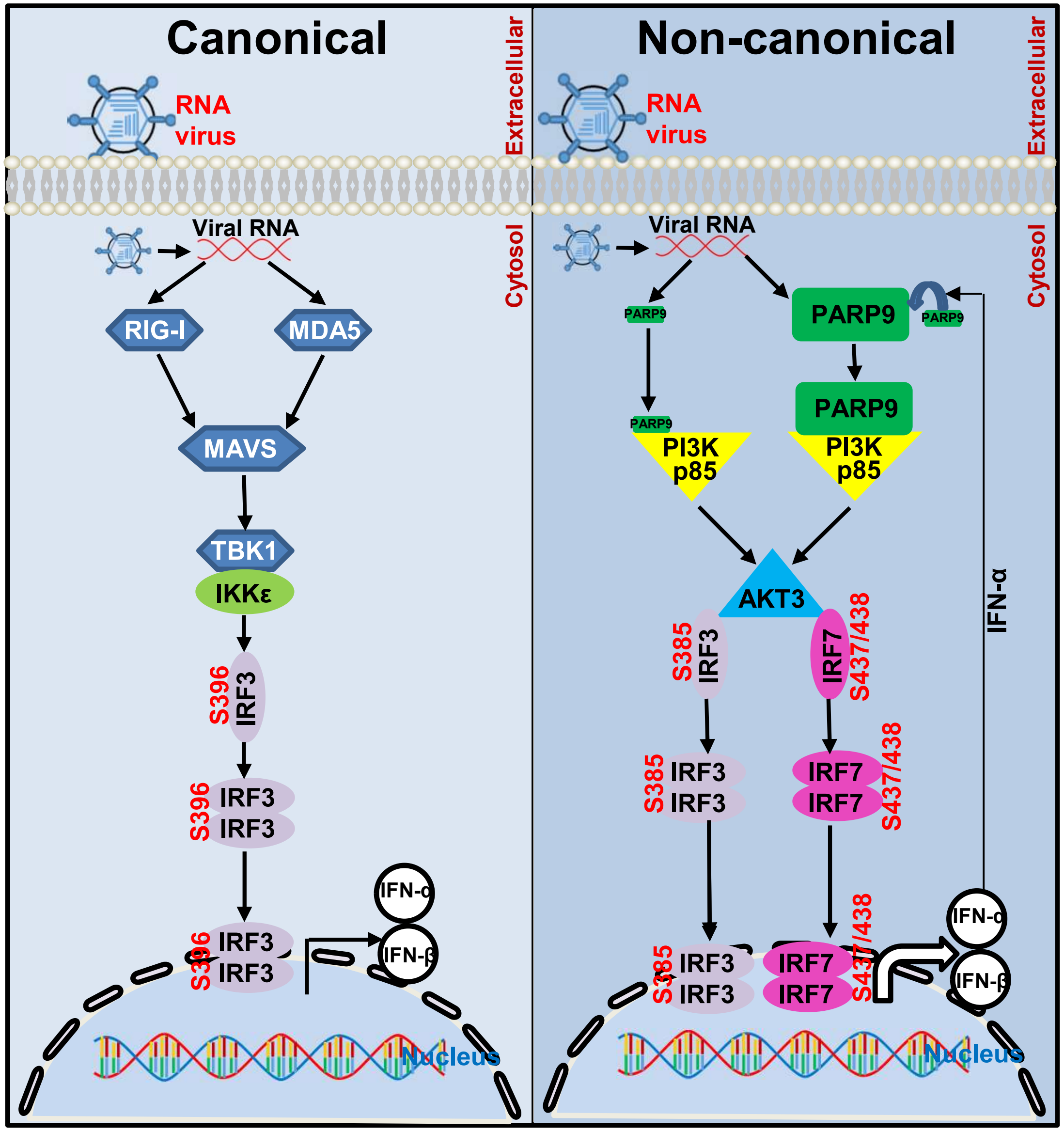

Supplementary Figure 14. Work model of PARP9 functions as non-canonical RNA sensor to induce MAVS-independent production of type I IFN. In canonical model, after RNA virus infection, well-known RNA sensors RIG-I and MDA-5 recognize viral RNA and induce MAVS-dependent type I IFN (IFN-α and IFN-β) production. In non-canonical model, upon RNA virus infection, the non-canonical RNA sensor PARP9 senses viral RNA and induces MAVS-independent type I IFN response by recruiting and activating PI3K p85 and AKT3 pathway. The produced IFN-α could induce dramatically PARP9 expression and the induced PARP9 further enhances the production of type I IFN and antiviral immune response.

Supplementary Table 1. mTOR is in the PARP9-binding protein complex in support of Fig. 6.

	Human anti-PARP9 no stimulation	
NCBI gi no.	Protein name description	Hits
15208660	E3 ubiquitin-protein ligase TRIM21 [Homo sapiens]	234
226371714	poly [ADP-ribose] polymerase 9 isoform b [Homo sapiens]	125
19923717	E3 ubiquitin-protein ligase DTX3L [Homo sapiens]	124
4504151	granulins precursor [Homo sapiens]	91
13654237	DNA-dependent protein kinase catalytic subunit isoform 1 (XRCC7) [Homo sapiens]	79
5453595	adenylyl cyclase-associated protein 1 [Homo sapiens]	34
10863927	peptidyl-prolyl cis-trans isomerase A [Homo sapiens]	30
4507677	endoplasmin precursor [Homo sapiens]	20
31377468	dedicator of cytokinesis protein 2 [Homo sapiens]	13
68509926	putative pre-mRNA-splicing factor ATP-dependent RNA helicase DHX15 [Homo sapiens]	12
4826730	serine/threonine-protein kinase mTOR [Homo sapiens]	12
11321601	6-phosphofructokinase type C isoform 1 [Homo sapiens]	11
144226251	chitinase-3-like protein 1 precursor [Homo sapiens]	11
5032179	transcription intermediary factor 1-beta [Homo sapiens]	11
100913206	ATP-dependent RNA helicase A [Homo sapiens]	10
102469694	galectin-9 isoform short [Homo sapiens]	10
6912286	caspase-14 precursor [Homo sapiens]	9
4504981	galectin-1 [Homo sapiens]	9
4503841	X-ray repair cross-complementing protein 6 [Homo sapiens]	9
9910382	mitochondrial import receptor subunit TOM22 homolog [Homo sapiens]	8
11024714	polyubiquitin-B precursor [Homo sapiens]	8
189458817	transferrin receptor protein 1 [Homo sapiens]	8
392513662	interleukin enhancer-binding factor 2 isoform 2 (NFAT 45) [Homo sapiens]	7
48255957	plasma membrane calcium-transporting ATPase 4 isoform 4b [Homo sapiens]	7
4502709	cyclin-dependent kinase 1 isoform 1 [Homo sapiens]	6
45446743	ATP-dependent RNA helicase DDX42 [Homo sapiens]	5
50659095	nucleolar RNA helicase 2 isoform 1 [Homo sapiens]	5
38201710	probable ATP-dependent RNA helicase DDX17 isoform 1 [Homo sapiens]	5
5031703	ras GTPase-activating protein-binding protein 1 [Homo sapiens]	5
21361399	serine/threonine-protein phosphatase 2A 65 kDa regulatory subunit A alpha isoform [Homo sapiens]	5
48255898	probable global transcription activator SNF2L2 isoform b [Homo sapiens]	4
392307009	receptor-type tyrosine-protein phosphatase C isoform 2 precursor [Homo sapiens]	4
6678271	TAR DNA-binding protein 43 [Homo sapiens]	4
5729770	tripeptidyl-peptidase 1 preproprotein [Homo sapiens]	4
21536301	signal transducer and activator of transcription 1(STAT1)-alpha/beta isoform beta [Homo sapiens]	3
193211480	superkiller viralicidic activity 2-like 2 [Homo sapiens]	3
68160937	E3 ubiquitin/ISG15 ligase TRIM25 [Homo sapiens]	2
295821162	TRAF3-interacting JNK-activating modulator [Homo sapiens]	2

40807469	TRAF-interacting protein [Homo sapiens]	2

Human THP-1 macrophages lysate was prepared, followed by anti-PARP9 immunoprecipitation and protein sequencing by liquid chromatography-mass spectrometry. NCBI gi no: unique protein identification number; Hits: the number of peptides ions matched that associated protein.

Supplementary Table 2. Primers for qRT-PCR and PCR used in this study.

Gene	Sequence
qRT-PCR	
Human *Parp9*	F: 5'- GGCAAAGAGGTCCAAGATGCTG -3'
	R: 5'- GCCTCACACATCTCTTCCACGT -3'
Human *Mavs*	F: 5'- GTGCCTACTAGCATGGTGCTC -3'
	R: 5'- GACCCAAGGCCCCTATTCT -3'
Human *Gapdh*	F: 5'- GAAATCCCATCACCATCTTCC -3'
	R: 5'- GAGCCCCAGCCTTCTCCATG -3'
Mouse *Ifn-β*	F: 5'-TCACCTACAGGGCGGACTTC -3'
	R: 5'-TCTCTGCTCGGACCACCATC -3'
Mouse *Gapdh*	F: 5'- AGGTCGGTGTGAACGGATTTG -3'
	R: 5'- TGTAGACCATGTAGTTGAGGTCA -3'
VSV	F: 5'- ACGGCGTACTTCCAGATGG -3'
	R: 5'- CTCGGTTCAAGATCCAGGT -3'
Mouse *Akt1*	F: 5'-ATGAACGACGTAGCCATTGTG-3'
	R: 5'-TTGTAGCCAATAAAGGTGCCAT-3'
Mouse *Akt2*	F: 5'-GGTCGCCAACAGTCTGAAG -3'
	R: 5'-TCTCTCGAACCAGAATGACCTT -3'
Mouse *Akt3*	F: 5'-TGGGTTCAGAAGAGGGGAGAA -3'
	R: 5'-AGGGGATAAGGTAAGTCCACATC -3'
Mouse *Parp9*	F: 5'-GCATTTGCTAAAGAGCACAAGGA -3'
	R: 5'-AAGCACCACTATTACCGCTGA -3'
Parp9 genotype PCR	
P1	5'-TCAGCTCTTGTAATGATGACTGC -3'
P2	5'-GAGAACGTGAGAAACTAAGACAGG -3'
P3	5'-GGGAGTCCTGGAATTAATGG -3'
P4	5'-GTATTCTAATCGCTTCTGGTTCC -3'
PCR	
Reo1187	F: 5'- TAATACGACTCACTATATTGCTTCTTCCCAGACGTTG -3'
	R: 5'-AAGCCTGTCCCACGTCACACCA -3'
Reo1198	F: 5'- TAATACGACTCACTATACCCCTGTCGTCGTCACTATGGC -3'
	R: 5'-GATGATTAGGCGTCACCCACCA -3'
Reo1320	F: 5'- TAATACGACTCACTATAGTTATGGCTCGCGCTGCGTTCCT -3'
	R: 5'-GGTCAGTCGTGAGGGGTGTGGGGGGACC -3'
Reo1410	F: 5'- TAATACGACTCACTATATCGGATGGATCCTCGCCTACGT -3'
	R: 5'-ATGCCCCAGTGCCGCGGGGTGGTC -3'
IRF3 S385A	F: 5'- CGGGTAGGGGGTGCCGCATCCCTGGAGAATACT -3'
	R: 5'- AGTATTCTCCAGGGATGCGGCACCCCCTACCCG -3'
IRF7 S483/484A	F: 5'- CTCAGCCTCTGCCTGGCAGCAGCCAACAGCCTCTAT -3'
	R: 5'- ATAGAGGCTGTTGGCTGCTGCCAGGCAGAGGCTGAG -3'
