# Supplementary material for: Identification of poly(ADP-ribose) polymerase 9 (PARP9) as a noncanonical sensor for RNA virus in dendritic cells
Source: Nat Commun. 2021 May 11;12:2681. doi: 10.1038/s41467-021-23003-4 (PMC8113569; doi:10.1038/s41467-021-23003-4)
Supplement: Supplementary file 3 — Reporting Summary [file 41467_2021_23003_MOESM3_ESM.pdf]

nature research

Corresponding author(s): Zhiqiang Zhang
Last updated by author(s): Mar 2, 2021

Reporting Summary

Nature Research wishes to improve the reproducibility of the work that we publish. This form provides structure for consistency and transparency in reporting. For further information on Nature Research policies, see our Editorial Policies and the Editorial Policy Checklist.

Statistics

For all statistical analyses, confirm that the following items are present in the figure legend, table legend, main text, or Methods section.

n/a	Confirmed	
☐	☒	The exact sample size (*n*) for each experimental group/condition, given as a discrete number and unit of measurement
☐	☒	A statement on whether measurements were taken from distinct samples or whether the same sample was measured repeatedly
☐	☒	The statistical test(s) used AND whether they are one- or two-sided<br>*Only common tests should be described solely by name; describe more complex techniques in the Methods section.*
☒	☐	A description of all covariates tested
☒	☐	A description of any assumptions or corrections, such as tests of normality and adjustment for multiple comparisons
☐	☒	A full description of the statistical parameters including central tendency (e.g. means) or other basic estimates (e.g. regression coefficient) AND variation (e.g. standard deviation) or associated estimates of uncertainty (e.g. confidence intervals)
☐	☒	For null hypothesis testing, the test statistic (e.g. *F*, *t*, *r*) with confidence intervals, effect sizes, degrees of freedom and *P* value noted<br>*Give P values as exact values whenever suitable.*
☒	☐	For Bayesian analysis, information on the choice of priors and Markov chain Monte Carlo settings
☒	☐	For hierarchical and complex designs, identification of the appropriate level for tests and full reporting of outcomes
☒	☐	Estimates of effect sizes (e.g. Cohen's *d*, Pearson's *r*), indicating how they were calculated

Our web collection on statistics for biologists contains articles on many of the points above.

Software and code

Policy information about availability of computer code

Data collection	ELISA: Synergy H1 Hybrid Multi-Mode Microplate Reader (BioTek INC),<br>q-RT-PCR: CFX96 Touch Real-Time PCR Detection System (Bio-Rad),<br>Luciferase reporter: GloMax 20/20 Luminometer (Promega Corporation),<br>Fluorescent images: Nikon A1 Confocal Microscopy (Nikon Corporation),<br>Western Blotting: SRX-101A Automatic X-ray Film Developer (Konica Minolta Medical &Graphic INC),<br>H&E staining images: EVOS M500 Cell Imaging System (Thermofisher),<br>DNA, RNA and protein concentrations: NanoDrop 2000 Spectrophotometer (Thermo Scientific).<br>Flow cytometry: FlowJo software version 10.6.0 (Tree Star Inc)
Data analysis	Data representation and statistical analysis: Graphpad Prism 8 and Microsoft Office Excel 2016. Immunofluorescence images were processed using NIS Elements Imaging Software5.11.02 (Nikon). Western blotting films were scanned by CanoScan 9000F Mark II. Images were processed with Adobe Photoshop Creative Cloud (CC) 2019 version20.0.10.

For manuscripts utilizing custom algorithms or software that are central to the research but not yet described in published literature, software must be made available to editors and reviewers. We strongly encourage code deposition in a community repository (e.g. GitHub). See the Nature Research guidelines for submitting code & software for further information.

Data

Policy information about availability of data

All manuscripts must include a data availability statement. This statement should provide the following information, where applicable:

- Accession codes, unique identifiers, or web links for publicly available datasets
- A list of figures that have associated raw data
- A description of any restrictions on data availability

All data generated or analyzed during the study are included in this published article and are available from the corresponding author upon reasonable request. The RNA sequencing (RNA-seq) data have been deposited with links to BioProject accession number "PRJNA609436 [https://www.ncbi.nlm.nih.gov/sra/PRJNA609436]" in the NCBI Sequence Read Archive (SRA) database. Source data are provided with this paper.

Field-specific reporting

Please select the one below that is the best fit for your research. If you are not sure, read the appropriate sections before making your selection.

[x] Life sciences [ ] Behavioural & social sciences [ ] Ecological, evolutionary & environmental sciences

For a reference copy of the document with all sections, see nature.com/documents/nr-reporting-summary-flat.pdf

Life sciences study design

All studies must disclose on these points even when the disclosure is negative.

Sample size	No statistical methods were used to predetermined sample size. Sample size was chosen by following the literature in the field.
Data exclusions	No data were excluded from the analyses.
Replication	All experimental findings were reliably reproduced in multiple independent experiments as indicated in the figure legends.
Randomization	We splitted mice equally and randomly allocated to each of experimental group in vivo according to their genotype.<br>Randomization was not relevant for other experiments, as they were performed in cell lines.
Blinding	The investigators were blinded to group allocation during data collection or analysis.

Reporting for specific materials, systems and methods

We require information from authors about some types of materials, experimental systems and methods used in many studies. Here, indicate whether each material, system or method listed is relevant to your study. If you are not sure if a list item applies to your research, read the appropriate section before selecting a response.

Materials & experimental systems

n/a	Involved in the study	
[ ]	[x]	Antibodies
[ ]	[x]	Eukaryotic cell lines
[x]	[ ]	Palaeontology and archaeology
[ ]	[x]	Animals and other organisms
[x]	[ ]	Human research participants
[x]	[ ]	Clinical data
[x]	[ ]	Dual use research of concern

Methods

n/a	Involved in the study	
[x]	[ ]	ChIP-seq
[ ]	[x]	Flow cytometry
[x]	[ ]	MRI-based neuroimaging

Antibodies

Antibodies used

Anti-PARP9 (IB:1:1000; IP: 1:100; AB10618, Millipore),
Anti-PARP9 (IB:1:1000; LS-B9440, LifeSpan BioScience),
Anti-PI3 kinase p85 alpha (IF:1:200; ab86714, Abcam),
anti-MAVS (IB:1:1000; sc-166583, Santa Cruz),
anti-IRF3 (IB:1:1000; sc-9082, FL-425, Santa Cruz),
anti-IRF7 (IB:1:1000; AHP1180T, Bio-Rad),
anti-phosphorylated IRF3 at Ser396 (IB:1:1000; #4947, Cell Signaling Technology),
anti-phosphorylated IRF3 at Ser385 (IB:1:1000; D151514, Sangon Biotech),
anti-phosphorylated IRF7 at Ser437/438 (IB:1:1000; #24129S, Cell Signaling Technology),
anti-p65 (IB:1:1000; #4764, Cell Signaling Technology),

anti-phosphorylated p65 (IB:1:1000; #3033, Cell Signaling Technology),
anti-mTOR (IB:1:1000; #2983S, Cell Signaling Technology),
anti-PI3 Kinase p85 (IB:1:1000; IP:1:100; #4257S, Cell Signaling Technology),
anti-TBK1 (IF:1:500, NB100-56705, Novus Biologicals),
anti-phosphorylated PI3 Kinase p85 (IB:1:1000; #4228S, Cell Signaling Technology),
anti-AKT1 (IB:1:1000; #2938S, Cell Signaling Technology),
anti-AKT2 (IB:1:1000; #3063S, Cell Signaling Technology),
anti-AKT3 (IB:1:1000; IP:1:100; #14982S, Cell Signaling Technology),
anti-phosphorylated AKT3 at Ser473 (IB:1:1000; #4060S, Cell Signaling Technology),
anti-β-actin (IB:1:10000; A3854, Sigma),
anti-HA (IB:1:5000; H6533, Sigma),
anti-Myc (IB:1:5000; ab1326, Abcam),
Peroxidase AffiniPure Goat Anti-Mouse IgG, light chain specific (IB:1: 10000, 115-035-174, Jackson ImmunoResearch),
Peroxidase AffiniPure Mouse Anti-Rabbit IgG, light chain specific (IB:1: 10000, 211-032-171, Jackson ImmunoResearch),
Alexa Fluor 488 goat anti-mouse secondary antibody (IF:1:1000; A-11001, ThermoFisher Scientific),
Alexa Fluor 594 goat anti-rabbit secondary antibody (IF:1:1000; A-11012, ThermoFisher Scientific),
APC/Cyanine7 anti-mouse CD45 Antibody (Clone: 30-F11; 103116, BioLegend),
FITC anti-mouse CD3 Antibody (Clone: 17A2; 100204, BioLegend),
PE/Cyanine7 anti-mouse CD4 Antibody (Clone: RM4-5; 100528, BioLegend),
APC anti-mouse CD19 Antibody (Clone: 1D3/CD19; 152410, BioLegend),
PerCP/Cyanine5.5 anti-mouse CD8a Antibody (Clone: 53-6.7; 100734, BioLegend)

Validation	All antibodies have been validated by the manufacturer. We only used antibodies recommended by the manufacturer for the species and application mentioned above.

Eukaryotic cell lines

Policy information about cell lines

Cell line source(s)	THP-1, L929, Vero and HEK 293T cell lines were purchased from ATCC. HEK 293FT cell lines were purchased from ThermoFisher Scientific.
Authentication	All cell lines were authenticated on their morphology and growth.
Mycoplasma contamination	All cell lines were tested and free of mycoplasma contamination.
Commonly misidentified lines (See ICLAC register)	No commonly misidentified cell lines were used.

Animals and other organisms

Policy information about studies involving animals; ARRIVE guidelines recommended for reporting animal research

Laboratory animals	he following inbred mouse species were used: C57BL/6J. Supplier and age (6-8 weeks) of the mice as well as housing conditions are provided for each experiment in the Methods section. Male and female mice were used. All animals were housed under 12h light/dark cycle at 22-24˚C with unrestricted access to food and water for the durations of the experiment.
Wild animals	The study did not involve wild animals.
Field-collected samples	No field collected samples were used.
Ethics oversight	The study is compliant with all relevant ethical regulations for animal experiments. All the experimental protocols were approved by the Houston Methodist Animal Care Committee, in accordance with institutional animal care and use committee guidelines.

Note that full information on the approval of the study protocol must also be provided in the manuscript.

Flow Cytometry

Plots

Confirm that:

- [x] The axis labels state the marker and fluorochrome used (e.g. CD4-FITC).
- [x] The axis scales are clearly visible. Include numbers along axes only for bottom left plot of group (a 'group' is an analysis of identical markers).
- [x] All plots are contour plots with outliers or pseudocolor plots.
- [x] A numerical value for number of cells or percentage (with statistics) is provided.

Methodology

Sample preparation	Preparation of cells from spleen was done by mechanical disaggregation of the tissue through a 100um strainer using a syringe plunger. Cell suspension was then moved to a collection tube and the strainer was washed twice with ice-cold PBS, followed by incubation in red blood cell lysis buffer (RBCLB) to remove the erythrocytes from the cell suspension. For each spleen, 1ml of room temperature RBCLB was added and the tube shaken manually for 2min before washing with ice-cold PBS. The cell suspensions was then used for flow cytometry.
Instrument	LSR-II flow cytometer (Beckton Dickinson)
Software	FACS data were analyzed using FlowJo v10 software (Tree Start).
Cell population abundance	No sorted samples were used.
Gating strategy	We have provided the gating strategy in Supplementary figure, providing all the necessary details. FSC-A/SSC-A was used to identify the lymphocytes by size. FSC-A/FSC-H followed by FSC-A/SSC-A were used to define singlets. Zombie Aqua fixable viability was used to identify live cells. APC/Cyanine7 anti-mouse CD45 antibody was used to identify CD45 positive cells. T cells were identified in spleen as CD3+ cells. CD4 and CD8 T cells were selected by the expression of CD4 and CD8, respectively. B cells were selected by the CD19+ CD3- expression.

☒ Tick this box to confirm that a figure exemplifying the gating strategy is provided in the Supplementary Information.
